# C-reactive protein (CRP) evaluation in human urine using optical sensor supported by machine learning

**DOI:** 10.1038/s41598-024-67821-0

**Published:** 2024-08-14

**Authors:** Kacper Cierpiak, Paweł Wityk, Monika Kosowska, Patryk Sokołowski, Tomasz Talaśka, Jakub Gierowski, Michał J. Markuszewski, Małgorzata Szczerska

**Affiliations:** 1 Department of Metrology and Optoelectronics, Faculty of Informatics, Telecommunications and Informatics, Gdańsk University of Technology, Narutowicza Street 11/12, 80-233 Gdańsk, Poland; 2 Department of Biopharmaceutics and Pharmacodynamics, Medical University of Gdańsk, Al. Gen. J. Hallera 107, 80-416 Gdańsk, Poland; 3 Department of Molecular Biotechnology and Microbiology, Chemical Faculty, Gdańsk University of Technology, 11/12 Narutowicza Street, 80-233, Gdańsk, Poland; 4 Faculty of Telecommunications, Computer Science and Electrical Engineering, Bydgoszcz University of Science and Technology, Al. prof. S. Kaliskiego 7, 85-796 Bydgoszcz, Poland; 5 Kayon sp. z o.o., Romualda Traugutta 115c, 80-226 Gdańsk, Poland; 6 Beckman Institute for Advanced Science and Technology, University of Illinois at Urbana-Champaign, Urbana, IL, USA

**Keywords:** Machine learning, C-reactive protein, Human urine, Fiber-optic sensors, Optical sensors, Optical sensors, Biomedical engineering, Machine learning

## Abstract

The rapid and sensitive indicator of inflammation in the human body is C-Reactive Protein (CRP). Determination of CRP level is important in medical diagnostics because, depending on that factor, it may indicate, e.g., the occurrence of inflammation of various origins, oncological, cardiovascular, bacterial or viral events. In this study, we describe an interferometric sensor able to detect the CRP level for distinguishing between no-inflammation and inflammation states. The measurement head was made of a single mode optical fiber with a microsphere structure created at the tip. Its surface has been biofunctionalized for specific CRP bonding. Standardized CRP solutions were measured in the range of 1.9 µg/L to 333 mg/L and classified in the initial phase of the study. The real samples obtained from hospitalized patients with diagnosed Urinary Tract Infection or Urosepsis were then investigated. 27 machine learning classifiers were tested for labeling the phantom samples as normal or high CRP levels. With the use of the ExtraTreesClassifier we obtained an accuracy of 95% for the validation dataset. The results of real samples classification showed up to 100% accuracy for the validation dataset using XGB classifier.

## Introduction

C-reactive protein (CRP) is an acute phase inflammatory protein being a sensitive and rapid indicator of inflammation in the body^1,2^. Inflammation is a nonspecific biological response of the immune system that can be triggered by harmful threats of various origins^3^.

CRP-level determination is an important diagnostic tool as it allows quick detection of inflammation, including bacterial infections^4^. In healthy adults, CRP level is typically < 3 mg/L^5,6^. However, in case of inflammation occurrence, it can increase up to a 1000-fold in few hours and peak within 2–3 days^7,8^. Increased CRP presence can be associated, depending on its level^5^ with oncological^9–11^, cardiovascular^12–14^, metabolic^15,16^, and bacterial or viral events^17,18^.

The gold standard for CRP determination is ELISA—enzyme-linked immunosorbent assay exhibiting high sensitivity, reaching pg/mL^19,20^. However, this method has several drawbacks: it is a time-consuming, expensive and complex procedure that has to be carried out by experienced personnel. Moreover, advanced biosensors are required for the measurements of samples with non-specific proteins^21^. These factors constitute the need for developing new solutions to enable reliable, fast, and cost-effective detection^22^. Moreover, interest in health monitoring and therapy support outside the hospitals^23^ due to progress in wearable electronics development^24,25^ justifies the usage of sensors of slightly worse metrological parameters but offering mobility, fast and user-friendly operation and potential for miniaturization.

Numerous scientific groups are conducting research to develop novel sensors to address this issue. One of the most promising approaches utilizes optical methods of detection due to their high sensitivity, fast response and non-destructive measurement manner. A label-free fiber optic biosensor based on surface plasmon resonance (SPR) was demonstrated^26^. A linear response was achieved for the CRP concentration ranging from 0.01 to 20 µg/mL. The sensor is compact and assures reliable results, the resonance wavelength shift proportional to the CRP level is recorded after 60 min. A. Aray et al. utilized an SPR-based plastic fiber-optic sensor for CRP detection in serum^27^. The measurement time was 15 min, with 20 µL of the sample required. An operating range of 6 µg/L to 70 mg/L was obtained, with a 9 µg/L detection limit. Another approach applies optical fiber Bragg gratings where CRP level determination is based on a Bragg wavelength^28^ shift. 200 µL of the sample was required for the measurements. The detection limit achieved was 0.01 mg/L, with a linear sensor response in the 0.01–100 mg/L range. Sensors with an optical cavity can also be applied in effective CRP detection^29^. The measurement time was below 30 min with a required sample volume of 15 µL. The detection limit achieved was 43.3 µg/L.

Our preliminary study proved that the fiber-optic sensor with a biofunctionalized measurement head is able to detect CRP levels in PBS solutions in a fast and reliable way^30,31^. It allows sample volume reduction, assuring a fixed cavity that increases stability and accelerates the measurement procedure, as well as allows for constant monitoring of the measurement head quality^32^.

In this research, we present a biophotonic sensor utilizing a biofunctionalized microsphere for CRP detection in human urine, and data classification using machine learning algorithms. The novelty of the proposed system lies in successful and reproduceable biochemical modification of a sensor made from commonly used telecommunication-grade glass fiber. The time from measurement to result reaches < 5 min which is a great improvement in comparison to ELISA protocols^33^. The innovative microsphere usage enables continuous monitoring of the probe integrity and biolayer state, desensitizing the probe to temperature or vibration impact. Furthermore, our trials have demonstrated the viability of this solution in urine testing, holding promise for online patient monitoring in hospitals when connected to a catheter. CRP levels measured for hospitalized patients with confirmed UTI or urosepsis are high. However, this might not be the case for patients in early inflammation stages, hence the need for viable classification of samples where only slight changes in CRP concentrations can occur. The sensor refers to challenging real human urine samples and the measurements were validated using gold standard—ELISA method. The developed binary sensor can be thus an effective support tool for pre-screening and early diagnosis.

## Materials and methods

### Measurement head functionalization

Prior to experiments on standardized CRP solutions, a measurement head has been biofunctionalized. The molecular sieves (3 Å, Pol-Aura, Poland) were activated in an oven (10 h, 350 °C) and cooled. Then they were transferred to the glass bottle with tight sealing and filled with dimethyl sulfoxide (DMSO; molecular biology grade, Merck, Germany) or acetone (molecular biology grade, Merck, Germany) (1:1 vol/vol). Solvents were then left for 72 h to dehydrate. The optical fiber with a microsphere was cleaned extensively by immersing it in H_2_O_2_/H_2_SO_4_ (WarChem, Poland; ACS reagent, Merck, Germany, respectively) water solution (10%, 50 mM respectively at 80 °C) for 30 min. After the cleaning process, the optical fiber was immersed in ultrapure water and finally in an anhydrous acetone solution to remove excess moisture. This step was repeated three times in different solutions, each incubation in an anhydrous environment took 10 min. So-prepared optical fiber was then immersed in a freshly prepared 1% 3-Aminopropyltriethoxysilane (APTES, 99% purity, Merck, Germany) solution in anhydrous acetone for 12 h at 70 °C in an anhydrous environment. to cover the optical fiber with amino groups. After 12 h the optical fiber was immersed in anhydrous DMSO (three times, 5 min of incubation) to remove excess APTES solution. Cleaned optical fiber with a microsphere covered with amino groups was then immersed in a freshly prepared 10 mM stock solution of NHS-LC-Biotin (95% purity, Merck, Germany) in anhydrous DMSO and left for 24 h in an anhydrous environment. After incubation, the optical fiber was immersed in the Streptavidin (1 mM solution, J&K Scientific, Poland) for 10 h at 15 °C. 1× PBS (phosphate buffered saline tablets; Merck, Germany) solution was then used to remove not immobilized proteins by extensive washing of the microsphere. Finally, the sensor was immersed in biotinylated anti-CRP Immunoglobulin G (IgG) (1 μg/μL, Merck, Germany) for 2 h, the excess of IgG was washed away by PBS solution. After the anti-CRP IgG attachment, a biological layer was formed on the surface of the microsphere.

### Measurement setup

**Figure 1:**
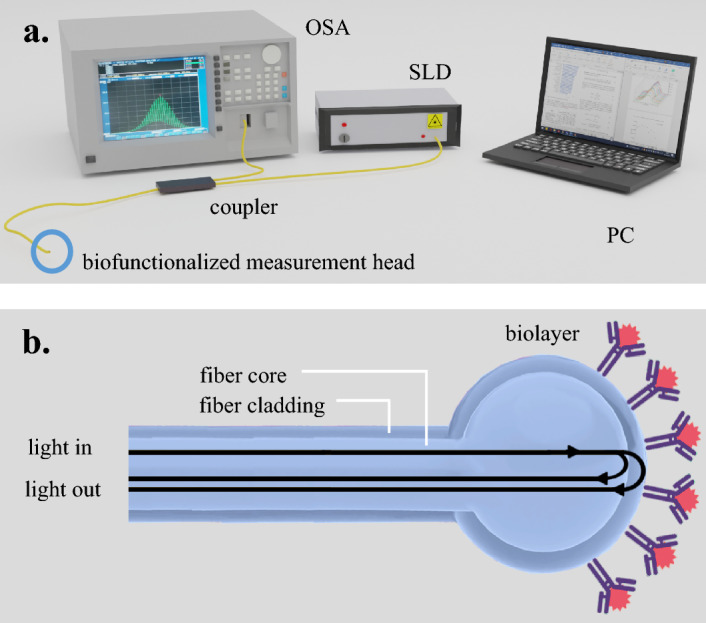
(**a**) Measurement setup: *SLD* SuperLuminescent Diode, *OSA* Optical Spectrum Analyzer. (**b**) The principle of measurement head operation.

The biophotonic measurement setup (Fig. 1a) was built of a broadband light source working at a central wavelength of 1310 nm (SLD-1310-18-W, FiberLabs Inc., Fujimi), a 2 × 1 fiber-optic coupler (G657A, CELLCO, Kobylanka, Poland) and an optical spectrum analyzer (Ando AQ6319, Yokohama, Japan). One of the coupler arms with a fiber connector was cut off and a single-mode optical fiber with a biofunctionalized microsphere was spliced to it. For the microsphere fabrication directly at the fiber tip, a standard telecommunication single mode optical fiber (SMF-28, Thorlabs, USA) and a fusion splicer (FSU 975, Ericsson Network Technologies AB, Stockholm, Sweden) were used. The microsphere (Fig. 1b) fabrication process was described in detail elsewhere^30,32,34,35^. Such probe design assures a fixed cavity (lowering impact of temperature and vibrations) and a real-time structure integrity monitoring, which in turn decreases the sensor cost, size, maintenance needs (no external mirrors and stabilizing micromechanical setups), simplifies probe placement in a measurement field, increases reliability and operation safety.

The principle of the sensor’s operation is based on interferometry. During the microsphere fabrication process, microsphere is created from optical fiber core coated by the cladding. The incident light guided through the optical fiber is split onto two waves which are reflected on created interfaces. A two-beam approximation can be applied: the first wave is reflected on the core/cladding boundary constituting a reference beam (independent of the measurand changes), and the second wave reflected on the cladding/measured medium boundary constitutes the measurement beam, which parameters are dependent on the measurand changes. The attachment of CRP to the microsphere surface changes its refractive index impacting the reflection coefficient, which allows for CRP level determination.

### Bioethics

The project plan was approved by the Bioethics Committee of the Medical University in Gdansk number: NKBBN/133/2019, prior to the initiation of the project. All patients or their authorized representatives provided written informed consent to participate in the project. All experiments were performed in accordance with the relevant guidelines and regulations.

## Results

The investigation included preparation, optical measurements and machine-learning classification of standardized CRP solutions. This phase was followed by the measurements and classification of the real samples, obtained from hospitalized patients. As such, two different datasets were created within this study: for standardized CRP level solutions in 1× Phosphate Buffer Saline (PBS) (283 spectra), and for real urine samples collected from patients (4382 spectra). The samples were also measured with an enzyme-linked immunosorbent assay (ELISA), a recognized standard method, to provide reference.

### Standardized CRP solutions

**Figure 2:**
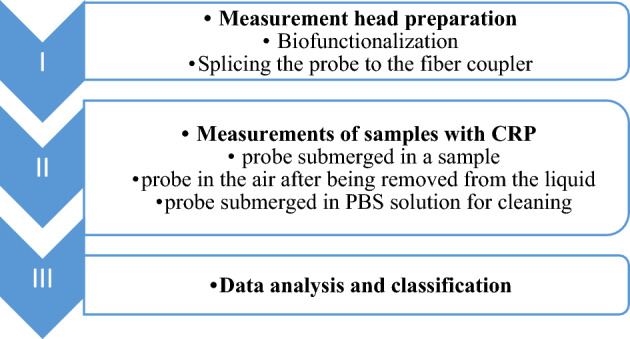
Experimental workflow scheme.

The experimental procedure for optical measurements with the developed sensor included three stages (Fig. 2): I—preparation of the measurement head by immersing the optical fiber in prepared solutions at 20 °C, II—series of measurements of the samples III—analysis of the obtained data and classification.

**Figure 3:**
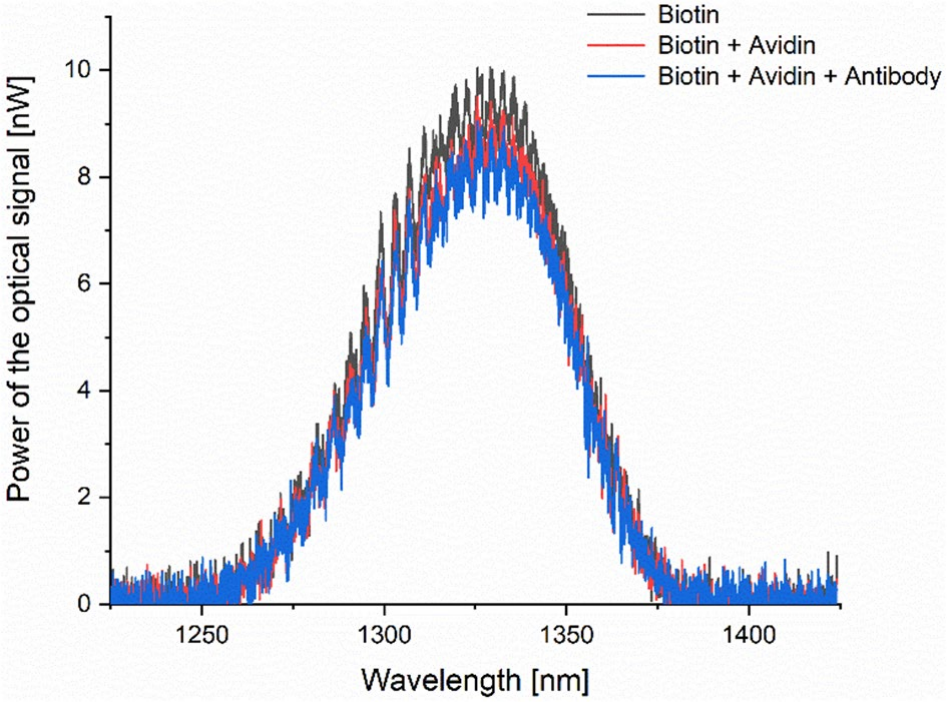
Optical spectra measured in PBS after covering the microsphere surface with consecutive layers, forming the biolayer specific for CRP.

During the preparation stage, the optical fiber with a microsphere was immersed in a series of liquids to biofunctionalize its surface for CRP detection. Successive immersions in Avidin and Antibody solutions resulted in the formation of layers on the surface of the microsphere. The optical spectra were recorded to monitor the impact of subsequent steps on the signal, as shown in Fig. 3. A visible change in the power of optical spectra occurs: its value decreases with the addition of consecutive layers. It can be explained by increasing absorption due to the attachment of molecules to the surface of the microsphere.

**Figure 4:**
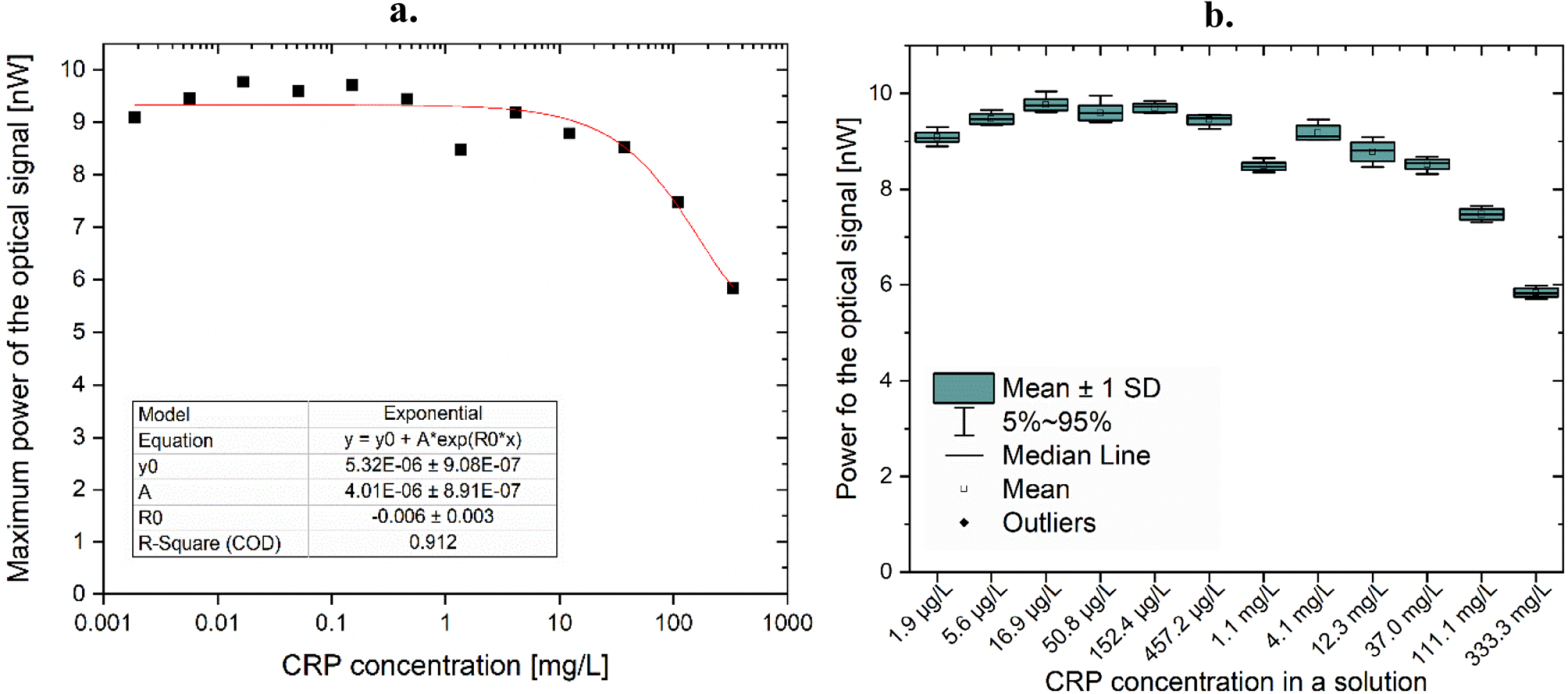
Measurement results for phantom samples (**a**) optical signal power as a function of CRP concentration, (**b**) box plots for each investigated sample.

The biofunctionalized measurement head was then used for measuring twelve CRP standardized solutions with CRP levels ranging from 333 mg/L to 1.9 µg/L. The measurement head was positioned in a micromechanical setup for convenient placing in the prepared liquid samples. The temperature of the samples was controlled throughout the whole experiment. Each sample was measured 10 times. The pilot study on the standardized CRP solutions ranging from 333 mg/L to 1.9 µg/L indicates viable results in the investigated range, both for low and high CRP levels. The work characteristics of the optical intensity sensor is exponential (Fig. 4a), the model fits the data with R^2^ = 0.912 (Fig. 4b).

The sensitivity of the instrument was presented for the hospitalized patients’ samples to validate its proper operation in the targeted circumstances. The outcomes were compared with the results obtained with the acknowledged standard—ELISA method. The investigation shows proper sensor operation and valid classification of the samples as normal or indicating inflammation. As the real samples were investigated, individual differences influence the range of measured CRP levels (i.e. the kind of inflammation, presence or absence of chronic diseases, age)—it ranged from 0.4 to 523 mg/L, the data represents in the majority more severe cases, but also milder and normal cases.

### Samples from patients

The methodology for CRP measurements in clinical samples involves a series of steps to ensure accurate and reliable results. First, urine samples were collected from individuals following established protocols: collecting the urine at least 10 mL, centrifugation (4000×*g*, 4 °C, 15 min), storage at − 80 °C and with appropriate ethical considerations (Bioethical committee agreement NKBBN/133/2019). The collection of urine samples took place at the University Clinical Medical Center in Gdansk. All patients or their authorized representatives provided written informed consent to participate in the project.

Inclusion criteria for diagnosing urosepsis based on the Sepsis 3.0 definition: (i)—Patient presenting to Emergency Department with suspicion of infection (ii)—*E. coli* present in urine and blood, observed as a culture and confirmed by genetics study (the same clinical strain found in urine and blood) (iii)—Patients eligible for the study were > 18 years old, diagnosed as urosepsis. (iv)—SIRS ≥ 2 (v)—Respiratory rate > 20/min or PaCO2 < 32 mmHg) (vi)—Heart rate > 90 bpm (vii)—Body temperature < 36 °C or > 38 °C (viii)—WBC < 4 × 10^9^/L or > 12 × 10^9^/L, > 10% immature neutrophils. Exclusion criteria: (i)—Sepsis other than urosepsis (ii)—Urosepsis of causative factors other than *E. coli* (iii)—Actively undergoing chemo- or radiotherapy due to cancer (iv)—Underage patients (v)—Lack of a patient's legal representative’s consent (vi)—Pregnancy and lactation (vii)—Hospital-acquired infection, (viii)—Antibiotic treatment in the last 2 weeks.

The CRP values obtained by ELISA, and qSOFA (quick Sequential Organ Failure Assessment) and SIRS (Systemic inflammatory response syndrome) parameters are listed in Table 1.

**Table 1:** Samples parameters.

Sample	Age	Sex	CRP [mg/L]	SIRS	qSOFA
1	46	f	324	0	0
2	70	m	75	0	1
3	88	f	147	0	2
4	72	m	313	2	1
5	61	f	34.3	0	0
6	70	f	35.5	0	0
7	73	f	130	1	0
8	73	f	296	0	0
9	25	f	43.5	2	2
10	66	m	33.5	1	0
11	52	m	40	2	0
12	58	m	144	1	0
13	31	f	255.6	2	0
14	20	f	50.4	1	0
15	62	m	200	0	0
16	81	m	141	0	0
17	79	m	210	2	1
18	47	f	111.4	1	0
19	55	m	140	2	0
20	77	m	228	1	1
21	30	m	73	2	1
22	77	f	101	1	2
23	81	f	180	0	0
24	86	f	156	0	0
25	41	m	113	2	0
26	29	f	277	2	0
27	66	m	165	0	0
28	69	f	160	2	0
29	69	m	523	2	2
30	88	m	0.4	1	0
31	85	f	48	no data	1
32	82	f	189.58	0	0
33	68	f	241	2	2
34	50	f	134	no data	0
35	66	m	148.31	2	0
36	68	m	24	2	0
37	85	f	181	3	0
38	71	f	243.56	0	0
39	57	f	412.38	0	1
40	85	m	58	0	0
41	88	f	11.32	0	0
42	84	m	104	0	0

To measure CRP levels in the clinical samples, a validated method ELISA was employed. These assays utilize specific antibodies that bind to CRP and produce a measurable signal, through fluorescent detection methods (Eppendorf Spectrofluorometer 2200, Germany was used for measurements). The assay (Human C-Reactive Protein ELISA Kit, Sigma Aldrich, Germany) was performed according to the manufacturer's instructions, including appropriate dilutions and incubation times. The samples were processed in duplicate to ensure accuracy and reproducibility. The CRP concentrations were then determined by comparing the sample readings to the standard curve, and the results were reported as mg/L. One sample was added from a patient that was not investigated by ELISA for intentional dataset noise generation.

**Figure 5:**
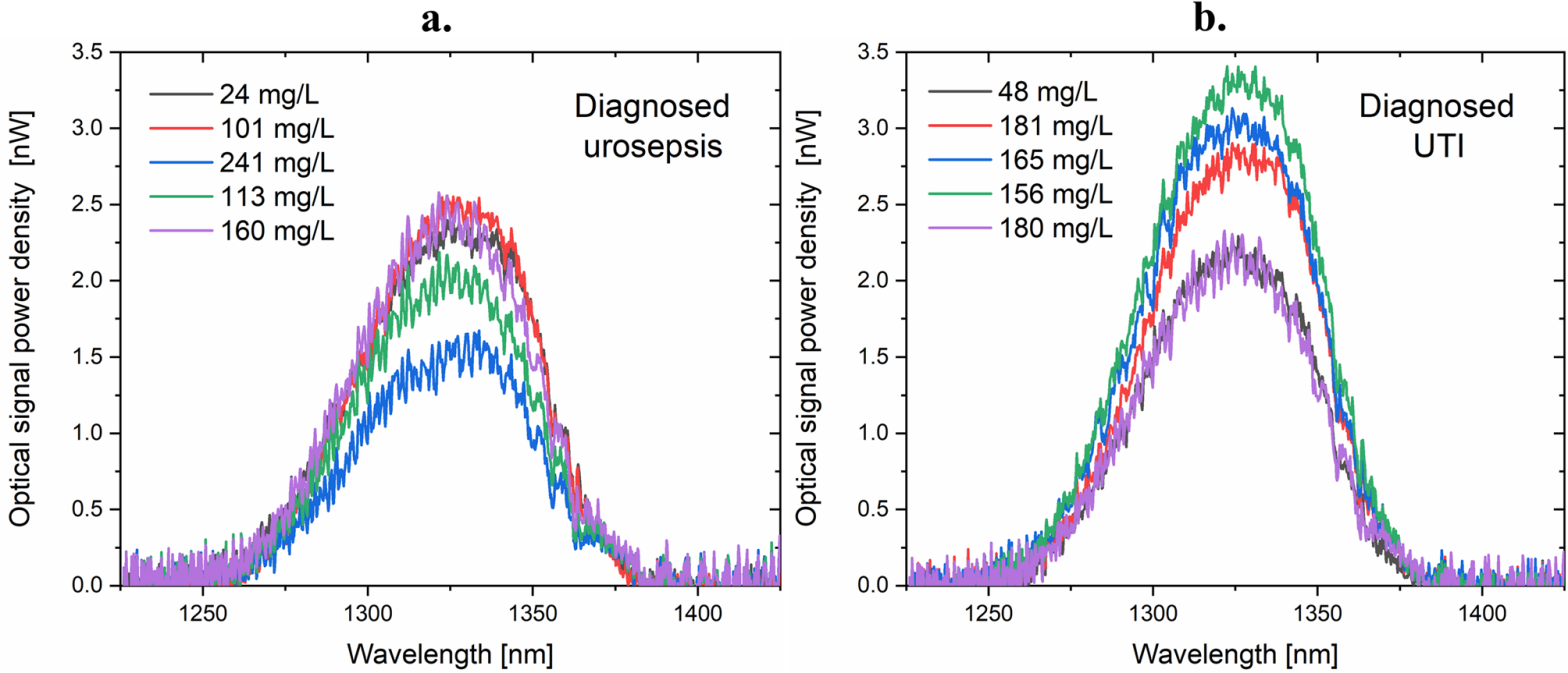
Representative optical signal for patients diagnosed with a. urosepsis or b. UTI. The legend shows the CRP level determined with the use of ELISA.

The samples were then measured with the developed interferometric sensor following the previously described procedure. 43 urine samples from patients with confirmed UTI (Urinary Tract Infection) or Urosepsis were investigated, each sample was measured ~ 100 times. The representative averaged optical spectra for real samples are presented in Fig. 5. In addition to the CRP level, other individual differences also affect the signal.

### Classification

The investigated algorithms and metrics description, as well as feature list and classification results for the chosen algorithms can be found in the Supplementary Material.

## Discussion

Proteins (antibodies/antigens), along with the structured water surrounding them, can contribute to the absorption of light around 1300–1600 nm^36^. Proteins (especially antibodies) are large biomolecules composed of amino acids, and they exhibit specific molecular structures and conformations. Certain chemical groups within proteins, such as amide bonds (found in the peptide backbone), can interact with light energy at specific wavelengths. In the near-infrared (NIR) region, which includes 1300–1600 nm, there are specific electronic transitions and vibrational modes within protein molecules that can cause absorption of light^37^.

Additionally, (and mostly) water molecules surrounding proteins can form what is often referred to as “structured water” or “hydration shells.” This structured water is influenced by the protein's surface and can have unique properties compared to bulk water (no bonding between antigen/antibody or no CRP in microsphere vicinity)^38^. The interaction between water molecules and the protein surface can result in changes in the water's hydrogen bonding network and molecular orientation. These alterations in the water structure can affect its absorption properties in the NIR region, including at 1300–1600 nm. At a wavelength of 1300–1600 nm, both proteins and structured water can contribute to the absorption of light^36^. The exact mechanisms depend on the specific protein structure, amino acid composition, and the arrangement of water molecules around the protein. Typically, the amide bonds and certain amino acid side chains within proteins, along with the altered hydrogen bonding patterns of structured water, can lead to absorption peaks in the NIR spectrum around 1310 nm. In summary, when CRP protein is absent there is less “solvation shell” around microsphere, thus the absorbance is lower (higher optical power observed), contrary when the antibody/antigen bond is form there is more protein absorption and structured water in solvation shell absorption resulting in lower optical signal.

As the density of CRP molecules binding to the microsphere increases, they tend to absorb more light, leading to a reduction in the detected light intensity. This decline in optical power can be directly correlated with heightened CRP levels in the sample, a relationship established through calibration via standard curves and previous experimental findings. Consequently, the observed attenuation in light intensity emerges as a reliable indicator of elevated CRP levels and, by extension, the presence of inflammation. It's noteworthy to recall that the antibody/antigen reaction stands as one of nature’s most specific and potent interactions, enabling antibodies to efficiently “capture” target protein molecules amidst a sea of other molecular entities. Leveraging this specificity and stability, akin to the gold standard enzyme-linked immunosorbent assay (ELISA) method^39^ in biochemistry, we opted to employ the antibody/antigen interaction alongside our optical fiber sensor. This decision ensures the specificity and robustness of our measurements.

Typically, CRP levels are gauged in blood samples; however, in instances of heightened inflammation, they can also manifest in urine. Ordinarily, the concentration of CRP in urine hovers around a range of 1–100 mg/mL, whereas in serum, it varies between 1 to 1000 mg/mL contingent upon the type of infection or the absence of inflammation^40^. Notably, the urinary CRP levels ought to be lower than those in serum.

However, in scenarios such as urosepsis or urinary tract infections (UTIs), urinary CRP levels may surge due to the presence of blood in urine or urine densification, a phenomenon often tied to creatinine levels (typically ranging from 0.7 to 1.3 mg/mL). Regrettably, the normalization of creatinine levels is often infeasible due to renal dysfunction during urosepsis or UTIs^41^. Hence, the changes in optical power shows a direct insight into the CRP concentration within the sample, thereby allowing the sensor to accurately discriminate between normal and elevated CRP levels associated with inflammatory conditions.

The obtained results indicate that the developed sensor can work as a binary sensor, allowing the classification of a tested CRP level as normal (No Inflammation) or high (Inflammation). The application of machine learning enables reliable decision-making in classifying the signals with increased accuracy in an efficient and fast way.

**Figure 6:**
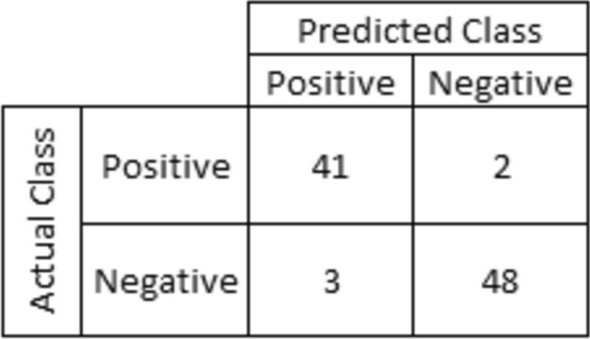
Confusion matrix for validation data (samples not involved in the learning process).

The test dataset contained 94 spectra. After performing prediction with the ExtraTrees, the algorithm classified 89 correctly, 5 incorrectly of which only 2 as false negatives Fig. 6.

The preliminary results made a strong basis for extending the investigation on real samples. The dataset consisted of 43 urine samples from patients with confirmed UTI (Urinary Tract Infection) or Urosepsis. Each sample was measured ~ 100 times, resulting in a total of 4382 spectra. As with phantom classification, the dataset was divided into two parts: 2/3 of the dataset was learning data and the remaining 1/3 was test data, which was used to validate the model. The division was made with the assurance that none measurement of the test samples was involved in the learning process. The preprocessing was the same as for the classification of the phantoms.

**Figure 7:**
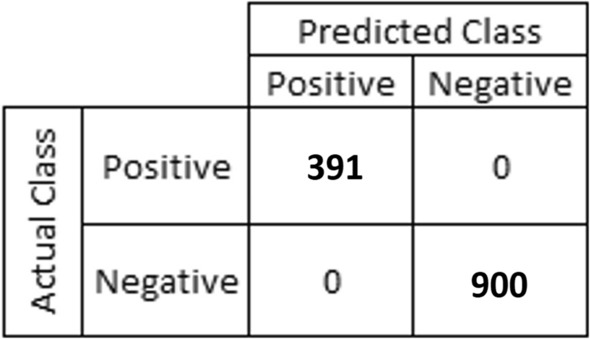
Confusion matrix for real data.

Based on the level of CRP, 2 classes were created: Moderate Elevation (ME) and Marked or Severe Elevation (MSE). The threshold value was taken as 10 mg/L^5^. The best results were obtained by the algorithm XGBClassifier with its Accuracy, AUC and F1 Score of 100%. Figure 7. presents a confusion matrix for validation data.

The high score achieved for accuracy results from preliminary study character of this research: samples from only 43 patients were used for dataset preparation. As such, we acknowledge that relatively small dataset may not be sufficient for general performance assessment. This issue will be resolved as we collect more samples and test the system on a larger and more diverse set of urine samples. However, for the purpose of a pilot study, we confirmed proper sensor operation and classification of real clinical samples from hospitalized patients, giving a strong basis for further system development. Regarding the sample investigated, its volume, measurement time and possibility of automated sample classification, selected methods described in the literature were compared to the described solution (Table 2).

**Table 2:** Comparison of the described method with current state-of-the-art.

No.	Method	Sample	Sample volume (μL)	Measurement time	Sample classification	References
1	Proposed solution	Human urine	10	< 5 min	Yes	N/A
2	CRP Human ELISA Kit	Plasma, serum, urine	5	80 min (time to result 4 h)	No	^42^
3	CRP Human Instant ELISA™ Kit	Plasma, serum, urine	10	20 min (time to result 2 h)	No	^43^
4	LPG in double cladding fiber coated with graphene oxide	Serum	40	20–30 min	No	^44^
5	SPR-based plastic optical fiber sensor	Serum	20	15 min	No	^27^
6	SPR-based CRP immunosensor	Serum	No data	30–60 min	No	^45^
7	Micromosaic immunoassays with self-regulating microfluidic networks	Plasma	1	10 min	No	^46^
8	Turbidimetric and Nephelometric Assays	Plasma, serum	5–20	5–15 min	No	^47^ ^48^
9	Immunoturbidimetric Assay	Plasma, serum	5–20	5–15 min	No	^49^
10	Latex Agglutination	Plasma, serum	5–30	10–30 min	No	^50^
11	Immunonephelometry	Plasma serum	100–200	30 min	No	^51^
12	Flow Cytometry	Serum, plasma, urine	10–200	1–3 h	No	^52^ ^53^

It must be noted that the developed measurement head can be reused but must be used with only one urine or human sample at a time. There is an urgent need for head change between samples because the diagnostic should be performed using a different sensor for each sample. Additionally, our study reveals that immersion of the sensor head in ultrapure water effectively removes the CRP protein bound to it, facilitating cleaning to some extent.

## Conclusion

This paper described a development of a binary biophotonic sensor for CRP detection. The sensor utilizes an optical fiber with a microsphere created on its end. The microstructure was biofunctionalized for the specific detection of CRP. The subsequent stages of layers deposition were optically monitored, confirming the proper coverage of the surface. The calibration curve for CRP levels ranging from 1.9 µg/L to 333 mg/L was obtained, with the sample volume of 10 µL required for the measurement. 27 classification algorithms were investigated to select the most efficient and effective from a computational complexity point of view for the sample labeling. The classification for real urine samples from patients with confirmed UTI or Urosepsis was performed on the basis of 4382 spectra. In case of real data, the best accuracy is proved by the XGBClassifier which can be as high as up to 100%. The results show high potential of the developed system as a supportive tool for pre-screening or early diagnosis.

### Supplementary Information

Supplementary Information.

## Data Availability

Data that support the findings of this study may be available upon request from the corresponding author.

## Code availability

Code used in this study may be available upon request from the corresponding author.
