# Supplementary material for: C-reactive protein (CRP) evaluation in human urine using optical sensor supported by machine learning
Source: Sci Rep. 2024 Aug 14;14:18854. doi: 10.1038/s41598-024-67821-0 (PMC11324656; doi:10.1038/s41598-024-67821-0)
Supplement: Supplementary file 1 — Supplementary Information. [file 41598_2024_67821_MOESM1_ESM.docx]

**SUPPLEMENTARY MATERIAL**

**C-Reactive Protein (CRP) evaluation in human urine
using optical sensor supported by machine learning**

Kacper Cierpiak^1^, Paweł Wityk^2,3^^, Monika Kosowska^4,^^, Patryk Sokołowski^1^, Tomasz Talaśka^4^, Jakub Gierowski^5^, Michał J. Markuszewski^2^, _,_ Małgorzata Szczerska^1,^*

*^1^ Department of Metrology and Optoelectronics, Faculty of Informatics, Telecommunications and Informatics, Gdańsk University of Technology, Narutowicza Street 11/12, 80-233, Gdańsk, Poland. e-mails: kacper.cierpiak@pg.edu.pl, patryk.sokolowski@pg.edu.pl, malszcze@pg.edu.pl*

*^2^ Department of Biopharmaceutics and Pharmacodynamics, Medical University of Gdańsk, Al. Gen. J. Hallera 107, 80-416 Gdańsk, Poland. e-mails: pawel.wityk@gumed.edu.pl, markusz@gumed.edu.pl*

*^3^ Department of Molecular Biotechnology and Microbiology, Chemical Faculty, Gdańsk University of Technology, 11/12 Narutowicza Street, 80-233, Gdańsk, Poland. e-mail: pawel.wityk@pg.edu.pl*

*^4^ Faculty of Telecommunications, Computer Science and Electrical Engineering, Bydgoszcz University of Science and Technology, Al. prof. S. Kaliskiego 7, 85-796 Bydgoszcz, Poland. e-mails: monika.kosowska@pbs.edu.pl, tomasz.talaska@pbs.edu.pl*

*^5^ Kayon sp. z o.o., Romualda Traugutta 115c, 80-226, Gdańsk, Poland. e-mail: jakub.gierowski@kayon.pl*

**Corresponding author: Małgorzata Szczerska - malszcze@pg.edu.pl*

*^^^ PW and MK have the same input in that work.*

1. ***Machine learning classifiers***

To be able to correctly describe the various metrics for the evaluation of classifiers, it is necessary to introduce the concepts of:

- True Positive (TP) – model correctly predicts the positive class, the sample labeled as inflammation was classified as inflammation.
- True Negative (TN) - model correctly predicts the negative class, the sample labeled as no inflammation was classified as no inflammation.
- False Positive (FP) - model incorrectly predicts the positive class. The sample labeled as no inflammation was classified as inflammation.
- False Negative (FN) - model incorrectly predicts the negative class. The sample labeled as inflammation was classified as no inflammation.

For the model that classifies CRP results, the most important matter is to minimize the occurrence of false negatives, which can cause a case of inflammation to be overlooked. The following metrics ^1^ are used for the evaluation of applied machine learning models.

- **Accuracy** is the basic and simplest metric of model quality it is the ratio between the quantity of correctly classified samples to the total quantity of samples, equation 1.

| $Accuracy= \frac{TP+TN}{TP+TN+FP+FN}$ | (1) |
| --- | --- |

- **Balanced Accuracy** separates negatively and positively classified samples, equation 2.

$\mathrm{Balanced}Accuracy= \frac{1}{2}\left( \frac{TP}{TP+FP}+\frac{TN}{TN+FN} \right)$ (2)

- **ROC AUC** Determines the possibility of a model to distinguish between positive and negative classes, AUC is the area under the ROC curve, the ROC curve is described by the equation 3 and 4.

$TPR= \frac{TP}{TP+FN}$ (3)

$FPR= \frac{FP}{FP+TN}$ (4)

- **F1 Score** determines the model's ability to recognize True Positive. It is built from two other metrics: Recall and Precision, equation 7. Recall measures the ability of the model to detect positive samples it is calculated from equation 5. Precision indicates what percentage is really positive of all positive predictions, equation 6.

| $Recall=TPR= \frac{TP}{TP+FN}$ | (5) |
| --- | --- |
| $Precision= \frac{TP}{TP+FP}$ | (6) |
| $F1 score= \frac{2\cdot(Precision\cdot Recall)}{Precision+Recall}$ | (7) |

To visualize the performance of an algorithm, a Confusion matrix is used, presenting a summary of TP, TN, FP and FN, as shown in Fig. S1.


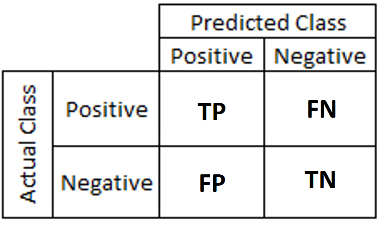


Fig. S1. Example of the confusion matrix

1. ***Machine learning algorithms applied in the investigation***

**2.1 Linear Models**

Linear models are a fundamental approach for classification. They establish a linear relationship between features and the target variable ^2–4^

- **LogisticRegression:** This workhorse algorithm calculates the probability of a data point belonging to a specific class. Great for interpretability and efficiency but struggles with non-linear relationships.
- **Perceptron:** A simple and interpretable algorithm for linearly separable data. Slow for large datasets and might not work for complex data.
- **SGDClassifier (Stochastic Gradient Descent Classifier):** A versatile tool that can be used for various classification tasks, including logistic regression. Efficient for large datasets but requires tuning hyperparameters for optimal performance.
- **RidgeClassifier & RidgeClassifierCV:** Regularized versions of Logistic Regression to prevent overfitting. RidgeClassifierCV automatically tunes a key parameter for better results.
- **PassiveAggressiveClassifier:** Similar to Perceptron, efficient for large datasets with linearly separable data. Updates weights only on mistakes, making it less prone to overfitting.
- **LinearSVC:** This is a linear variant of the Support Vector Machine algorithm. Efficient for high-dimensional data but limited to linear decision boundaries.

**2.2 Naive Bayes**

Naive Bayes classifiers are probabilistic models based on Bayes' theorem. They assume independence between features. They calculate the probability of a data point belonging to a class based on individual feature probabilities. which might not always hold true in real data. However, they can be efficient for specific problems due to their simplicity ^3,5^.

- **BernoulliNB:** This variant is suitable for binary classification problems with Boolean features (True/False). In our case we are mapping features to booleans with threshold (binarizing)
- **GaussianNB:** This version assumes features follow a Gaussian (normal) distribution.

**2.3 Support Vector Machines (SVM)**

SVMs aim to find a hyperplane in the feature space that maximizes the margin between the different classes. They are powerful for high-dimensional data and can handle non-linear data through kernel methods ^3,5^.

- **SVC (Support Vector Classifier):** This is the most common SVM implementation.
- **NuSVC:** This variant uses a parameter 'nu' to control the number of data points allowed on the wrong side of the margin, offering more flexibility.

**2.4 Nearest Neighbors**

Nearest Neighbors algorithms classify data points based on the class of their closest neighbors in the feature space ^3,5^.

- **KNeighborsClassifier:** This method classifies a data point based on the majority vote of its k nearest neighbors.
- **Nearest Centroid:** This simpler approach classifies a data point based on the closest centroid (average point) of its class in the feature space.

**2.5 Tree-based Algorithms**

These algorithms make classification decisions based on a tree-like structure. Data points are split based on features at each node, leading to a final leaf node representing a class ^3,5^.

- **DecisionTreeClassifier:** This is a fundamental tree-based algorithm that splits data points based on thresholds in their features.
- **ExtraTreeClassifier:** An extra-tree is a kind of decision tree used in machine learning. It's similar to a regular decision tree, but with more randomness in how it splits data. This can help improve accuracy and reduce overfitting.
- **ExtraTreesClassifier & RandomForestClassifier:** These ensemble methods build multiple decision trees and aggregate their predictions for improved accuracy and robustness. ExtraTreesClassifier is similar to Random Forest but uses random splitting at each node, while Random Forest considers the best split among a random subset of features.

**2.6 Boosting Algorithms**

Boosting algorithms iteratively build an ensemble of weak learners (in our case decision trees). Each subsequent learner focuses on the data points that the previous ones misclassified, leading to a more robust final model ^3,5^.

- **AdaBoostClassifier (Adaptive Boosting):** This is a classic boosting algorithm that assigns weights to data points, focusing on the ones that were previously misclassified.
- **XGBClassifier (Extreme Gradient Boosting):** XGBoost is a powerful and widely used boosting algorithm known for efficiency, scalability, and handling complex interactions between features.
- **LGBMClassifier (Light Gradient Boosting Machine):** Similar to XGBoost, LGBM is another popular boosting algorithm known for speed and efficiency.

**2.7 Discriminant Analysis**

These methods assume a Gaussian distribution for each class and aim to find a linear transformation to maximize the separation between classes. They project data points onto a lower-dimensional space for classification ^4,5^.

- **Quadratic Discriminant Analysis (QDA):** Allows for curved decision boundaries, potentially better capturing complex class separations, but can be prone to overfitting.
- **Linear Discriminant Analysis (LDA):** Limited to linear decision boundaries, but computationally efficient and can be a good choice for high-dimensional data.

**2.8 Bagging Algorithms**

Bagging (bootstrap aggregating) is another ensemble method that trains multiple models on different subsets of the data with replacement, leading to improved predictions ^3,5^.

- **BaggingClassifier:** This is a general implementation of bagging that can be used with various base models, in our case decision trees.

**2.9 Other Algorithms**

This category includes various classification algorithms that don't neatly fit into the above categories ^3,5^.

- **CalibratedClassifierCV:** This is a wrapper that calibrates the probabilities provided by any other classification model, improving their reliability.
- **LabelPropagation & LabelSpreading:** These algorithms are used for semi-supervised learning, where a small amount of labeled data is used to classify a larger set of unlabeled data.
- **DummyClassifier:** This is a simple classifier that serves as a baseline for comparison. It can be used to predict the most frequent class or employ a random classification strategy.

***References***

1. Yadavendra & Chand, S. A comparative study of breast cancer tumor classification by classical machine learning methods and deep learning method. *Machine Vision and Applications* **31**, 46 (2020).

2. James, G., Witten, D., Hastie, T. & Tibshirani, R. *An Introduction to Statistical Learning: With Applications in R*. (Springer, New York Heidelberg Dordrecht London, 2013).

3. Géron, A. *Hands-On Machine Learning with Scikit-Learn, Keras, and TensorFlow: Concepts, Tools, and Techniques to Build Intelligent Systems*. (O’Reilly Media, Beijing China ; Sebastopol, CA, 2019).

4. Bishop, C. M. *Pattern Recognition and Machine Learning (Information Science and Statistics)*. (Springer-Verlag, Berlin, Heidelberg, 2006).

5. Pedregosa, F. *et al.* Scikit-learn: Machine Learning in Python. *J. Mach. Learn. Res.* **12**, 2825–2830 (2011).

1. ***Features used in signal classification***

Table S1. Features used in signal classification for measured optical spectra.

| Feature | Description |
| --- | --- |
| All_PowerSpectrum | Sum of the power density over the full measured range |
| All_Amean | Arithmetic mean of the power density over the full measured range |
| All_Gmean | Geometric mean of the power density over the full measured range |
| All_Hmean | Harmonic mean of the power density over the full measured range |
| All_Median | Median power density over the full measured range |
| All_AUC | Area under the curve (AUC) of power density over the full measured range |
| All_Entropy | Entropy of power density over the full measured range |
| All_MAD | Median absolute deviation (MAD) of power density over the full measured range |
| All_Min | Power density minimal value over the full measured range |
| All_Max | Power density maximal value over the full measured range |
| All_Var | Power density variance value over the full measured range |
| All_Std | Power density standard deviation value over the full measured range |
| All_Skewness | Power density skewness over the full measured range |
| All_Kurtosis | Power density kurtosis value over the full measured range |
| All_Centroid | Power density centroid value over the full measured range |
| Range_PowerSpectrum | Sum of the power density over the range between first and last peak |
| Range_Amean | Arithmetic mean of the power density over the range between first and last peak |
| Range_Gmean | Geometric mean of the power density over the range between first and last peak |
| Range_Hmean | Harmonic mean of the power density over the range between first and last peak |
| Range_Median | Median power density over the range between first and last peak |
| Range_AUC | Area under the curve (AUC) of power density over the range between first and last peak |
| Range_Entropy | Entropy of power density over the range between first and last peak |
| Range_MAD | Median absolute deviation (MAD) of power density over the range between first and last peak |
| Range_Min | Power density minimal value over the range between first and last peak |
| Range_Max | Power density maximal value over the range between first and last peak |
| Range_Var | Power density variance value over the range between first and last peak |
| Range_Std | Power density standard deviation value over the range between first and last peak |
| Range_Skewness | Power density skewness over the range between first and last peak |
| Range_Kurtosis | Power density kurtosis value over the range between first and last peak |
| Range_Centroid | Power density centroid value over the range between first and last peak |
| WL_Min | Positon of first peak |
| WL_Max | Position of last peak |
| WL_Mean | Mean of peaks positions |
| WL_Size | Distance between first and last peak |
| Peaks_Qty | Number of peaks detected in the signal |
| Peaks_P_Min | Peaks prominence minimal value |
| Peaks_P_Max | Peaks prominence maximal value |
| Peaks_P_Mean | Peaks prominence arithmetic mean |
| Peaks_P_Std | Peaks prominence standard deviation |
| Peaks_P_Var | Peaks prominence variance |
| Peaks_W_Min | Peaks width minimal value |
| Peaks_W_Max | Peaks width maximal value |
| Peaks_W_Mean | Peaks width arithmetic mean |
| Peaks_W_Std | Peaks width standard deviation |
| Peaks_W_Var | Peaks width variance |
| Peaks_WH_Min | Peaks width heights minimal value |
| Peaks_WH_Max | Peaks width heights maximal value |
| Peaks_WH_Mean | Peaks width heights arithmetic mean |
| Peaks_WH_Std | Peaks width heights standard deviation |
| Peaks_WH_Var | Peaks width heights variance |
| Peaks_D_Min | Distance between peaks minimal value |
| Peaks_D_Max | Distance between peaks maximal value |
| Peaks_D_Mean | Distance between peaks arithmetic mean |
| Peaks_D_Std | Distance between peaks standard deviation |
| Peaks_D_Var | Distance between peaks variance |

1. ***Classification***

Series of measurements of the phantoms with known CRP concentrations were performed for each sample. The dataset consisted of a total of 283 spectra. The dataset was divided into two parts: 2/3 of the set was training data, and the remaining 1/3 was test data which were used to validate the model. The data preprocessing phase included de-noising the spectra using a low-pass filter, feature engineering and selection. 57 features relevant to the CRP classification problem were determined based on the optical spectra, for example., maximum, mean, standard deviation, kurtosis, and skewness of the optical signal. The complete list of the metrics can be found in Table S1.

Based on the level of CRP, 2 classes were created: Inflammation, and No Inflammation. The threshold value was taken as 10 mg/L. Twenty-seven classification algorithms were analyzed, and the accuracy results of each algorithm are presented in Fig.S2.


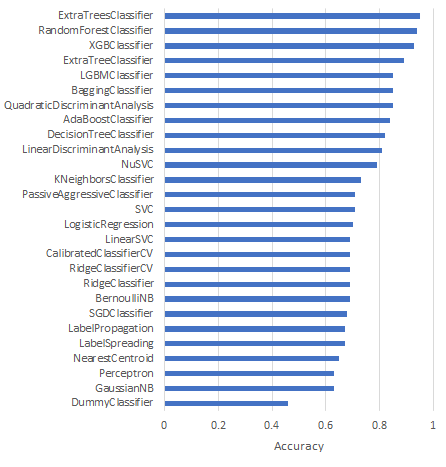


Fig. S2 Classification accuracy for specific algorithm.

All results obtained are for the validation dataset. For the top 5 algorithms in terms of accuracy, the quality metrics released: Balanced Accuracy, ROC AUC, F1 and Time Taken [s] are shown in Table S2. ExtraTreesClassifier algorithm achieved the best results for quality indicators.

Table S2. Detailed results of the top 5 models.

| **Model** | **Accuracy** | **Balanced Accuracy** | **ROC AUC** | **F1 Score** | **Time Taken [s]** |
| --- | --- | --- | --- | --- | --- |
| ExtraTreesClassifier | 0.95 | 0.95 | 0.95 | 0.95 | 0.14 |
| RandomForestClassifier | 0.94 | 0.94 | 0.94 | 0.94 | 0.24 |
| XGBClassifier | 0.93 | 0.92 | 0.92 | 0.93 | 0.12 |
| ExtraTreeClassifier | 0.89 | 0.90 | 0.90 | 0.89 | 0.01 |
| QuadraticDiscriminationAnalysis | 0.85 | 0.85 | 0.85 | 0.85 | 0.03 |

The biological samples, obtained from a substantial patient population, demonstrate notable disparities in the outcomes of the individual algorithms when compared to the phantom measurements. These phantom measurements were conducted as a vital means to validate the sensor's performance, thereby establishing a compelling rationale for the utilization of biological samples.
